# Data Augmentation Using Adversarial Image-to-Image Translation for the Segmentation of Mobile-Acquired Dermatological Images

**DOI:** 10.3390/jimaging7010002

**Published:** 2020-12-24

**Authors:** Catarina Andrade, Luís F. Teixeira, Maria João M. Vasconcelos, Luís Rosado

**Affiliations:** 1 Fraunhofer Portugal AICOS, Rua Alfredo Allen, 4200-135 Porto, Portugal; maria.vasconcelos@fraunhofer.pt (M.J.M.V.); luis.rosado@fraunhofer.pt (L.R.); 2 Faculty of Engineering, University of Porto, Rua Dr. Roberto Frias, 4200-465 Porto, Portugal; luisft@fe.up.pt; 3 INESC TEC, Rua Dr. Roberto Frias, 4200-465 Porto, Portugal

**Keywords:** convolutional neural network, CycleGAN, data augmentation, dermoscopic images, domain transfer, macroscopic images, skin lesion segmentation

## Abstract

Dermoscopic images allow the detailed examination of subsurface characteristics of the skin, which led to creating several substantial databases of diverse skin lesions. However, the dermoscope is not an easily accessible tool in some regions. A less expensive alternative could be acquiring medium resolution clinical macroscopic images of skin lesions. However, the limited volume of macroscopic images available, especially mobile-acquired, hinders developing a clinical mobile-based deep learning approach. In this work, we present a technique to efficiently utilize the sizable number of dermoscopic images to improve the segmentation capacity of macroscopic skin lesion images. A Cycle-Consistent Adversarial Network is used to translate the image between the two distinct domains created by the different image acquisition devices. A visual inspection was performed on several databases for qualitative evaluation of the results, based on the disappearance and appearance of intrinsic dermoscopic and macroscopic features. Moreover, the Fréchet Inception Distance was used as a quantitative metric. The quantitative segmentation results are demonstrated on the available macroscopic segmentation databases, SMARTSKINS and Dermofit Image Library, yielding test set thresholded Jaccard Index of 85.13% and 74.30%. These results establish a new state-of-the-art performance in the SMARTSKINS database.

## 1. Introduction

Skin cancer is one of the most prevalent malignancy worldwide and has a reported yearly growing incidence with no signs of plateauing. In 2018, almost three-hundred thousand malignant melanoma (MM) were diagnosed worldwide and over a million non-melanoma skin cancers (NMSC) [1]. However, this number excludes basal cell carcinoma, the most frequent skin cancer, since most go unreported [2]. The majority of NMSC and MM are highly curable if diagnosed in the early stages. The estimated 5-year survival rate for MM drops from over 98% to 23% if detected when the metastases are distant from the origin point [1]. Moreover, the effective diagnosis is compromised by the almost identical clinical presentation of benign and malignant skin lesions. Therefore, timely and accurate diagnosis is a paramount measure in controlling this pre-eminent global public health problem.

The growing usage of smartphones added to the robustness of deep learning models makes a mobile-based deep learning approach a well-suited possibility for the automatic cutaneous cancer triage [3,4]. Notwithstanding, the limited volume of mobile acquired images, such as macroscopic or close-up images, proves to be an obstacle in the development of a robust model specific to the macroscopic image type. Dermoscopy is the standard procedure for skin cancer preliminary diagnosis, it allows the visualization of inner layers of the skin which are not visible to the naked eye. This visualization method added to the procedural algorithms, allowed an expert diagnostic accuracy of 75% to 84% [5,6,7], which compelled the generation of several sizeable databases of this type of images. Yet, the direct inference between the two domains, macroscopic and dermoscopic, is not advisable and may even prove to be a detriment for the robustness of the model [8,9]. These image acquisition formats generate images with very different characteristics and challenges [10] and even for the clinical diagnosis, there are rules and methods specific for each domain [11].

This work aims to evaluate the possibility of designing a deep learning algorithm for the precise segmentation of lesion in macroscopic images capable to fully operate in the mobile environment. To assemble such a model, we explore the capitalization of the sizable dermoscopic databases by using a Cycle-Consistent Adversarial Network (CycleGAN) [12] for the translation between the two domains, macroscopic and dermoscopic.

## 2. Related Work

One of the fundamental challenges of the medical imaging computer vision analysis is to achieve satisfactory results with limited labelled datasets. Even in the era of Big data, there are still some challenges for which the amount of available data is still severely lacking. This is the case for the macroscopic segmentation challenge.

The lack of macroscopic images may be the reason for the few efforts into developing segmentation algorithms [10,13,14,15,16,17,18]. Examples of segmentation techniques used are—thresholding [14,16,17], unsupervised dictionary learning methods [13] and support vector machines [15]. Rosado et al. [16] used an adaptive thresholding technique with strong pre and post-processing techniques. The usage of deep learning methods has also been reported [10,18]. In Fernandes et al. [18], a convolution neural network (CNN), with gossip blocks, combined with a backpropagation technique was used to improve the segmentation masks by maximizing the expected quality. In the previous work [10] several architectural modifications of two widely used segmentation CNNs, U-Net and DeepLab, were tested to infer the best macroscopic segmentation CNNs. Here, two methodologies were tested to attempt to mitigate the small size of the macroscopic databases: heavy classical augmentation and transfer learning. The highest reported segmentation performance obtained, in a macroscopic database, was in Reference [10] with an 82.64% Jaccard index.

One of the techniques used to lessen the small datasets is data augmentation through basic image manipulation such as flipping, rotating and addition of noise [10,18]. However, with very small datasets extensive classical data augmentation may promote overfitting [19]. Another strategy used for data augmentation is the creation of artificial instances through generative adversarial networks. This type of framework samples from simple distributions and learn to transform it into the complex high dimensional distributions. For this purpose, two networks are used: one generating network which creates real-looking images, and a discriminator network which tries to distinguish between the real and fake images. Several techniques have been attempted for the skin lesion generation [20,21,22,23] however most are used for data augmentation applied to classification task, or to generate skin lesions from random noise without little to no prior knowledge of the complexity of the lesion.

More recently, Gu et al. [9] used a CycleGAN to perform cross-domain skin diseases classification between different disease databases. The CycleGAN algorithm performs image-to-image translation without the need for paired training samples, where the referred work proved the suitability of this approach to reduce the domain shift between different datasets. Thus, in this paper, we propose the CycleGAN to transform the dermatoscopic images into macroscopic. This transformation would allow the use of the vast amount of available dermatological images to increase the robustness of approaches in the macroscopic domain, while maintaining structural information of the lesions and some of their characteristics.

## 3. Materials and Methods

### 3.1. Databases

The growing interest in computer-aided diagnosis systems for automated detection and classification of skin lesions led to the creation of several databases. Most of these have been used by researchers to develop algorithms for automated segmentation of lesion borders.

#### 3.1.1. Segmentation Databases

**Macroscopic Segmentation Databases** Although many databases provide matching binary segmentation masks of cutaneous lesions only the Dermofit image Library [24] and the SMARTSKINS database [25] are of the macroscopic domain. The Dermofit digital image database consists of 1300 high-quality colour skin lesions images taken with standard cameras–with matching binary segmentation masks and class labels, the lesions belong to 10 different diseases with 819 benign and 481 carcinogenic images, annotated by individual disease classes. The SMARTSKINS database was obtained at the Skin Clinic of the Portuguese Institute of Oncology of Porto [25]. This database was acquired with mobile devices and it comprises several subsets captured in different years. One of the subset consists of 80 melanocytic lesions together with the corresponding segmentation masks, as well as medical annotations regarding ABCD score and overall risk.

**Dermoscopic Segmentation Databases** In terms of dermoscopic databases, we used the three publicly available International Skin Imaging Collaboration (ISIC) challenges datasets (2016 [26], 2017 [27] and 2018 [28,29]), as well as the PH2 database [30]. The ISIC database is a collection of multiple databases used in a recurrent challenge, which aims to improve melanoma detection in dermoscopic images. The ISIC Challenge is broken into 3 tasks—lesion segmentation, lesion attribute detection and disease classification. The PH2 database was obtained at the Dermatology Service of Hospital Pedro Hispano. Each case contains a dermoscopic image, the segmentation of the lesion provided by a doctor, as well as clinical and histological diagnosis and the assessment of several dermoscopic criteria.

**Figure 1:**
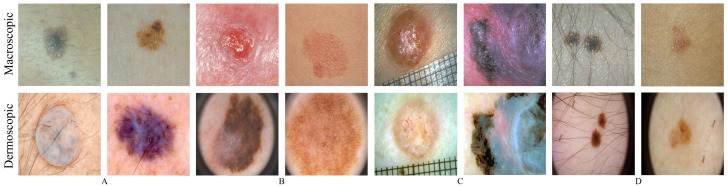
Illustrative examples of macroscopic (row above) and dermoscopic (below) skin lesions. (**A**) above: SMARTSKINS (Set M); below: ISIC (set D); (**B**) above: Dermofit (Set M); below: PH2 (set D); (**C**) EDRA; (**D**) SMARTSKINS 2014/2015.

The splitting process performed on these databases is summarized in Table 1, which is the same used in Reference [10]. Essentially, the Dermofit and PH2 databases suffer an 80/20 split, between the train and test subsets, and the SMARTSKINS a 50/50 split due to its relative small size. For the three ISIC challenges, the images were merged and the ISIC 2017 test instances were reserved for the test subset. Figure 1 shows illustrative examples of the macroscopic and dermoscopic segmentation databases in pairs A and B.

#### 3.1.2. Macroscopic and Dermoscopic Databases

The particular characteristic of these databases is that for each skin lesion it has both macroscopic and dermoscopic image. Two databases were found that enter this category—the EDRA database [31] and a subset of the SMARTSKINS database [25]. Figure 1 shows illustrative examples of the two aforementioned databases in pairs C and D, each column has above the macroscopic images and below the dermoscopic counterpart. The EDRA database comprises over 1000 cases of skin lesions. Each clinical case is properly annotated with the clinical data, histopathological results, diagnosis, level of difficulty and the macroscopic and dermoscopic image of the lesion. The subset SMARTSKINS database (SMARTSKINS 2014/2015) was acquired with two smartphones and classified by the overall risk of the lesions. This subset contained over 170 cases of lesions, each of them had one dermoscopic image and two macroscopic.

These databases were used to form the macroscopic and dermoscopic dataset. The division of these databases, into train and test subsets, was performed in a stratified manner in accordance with the classification. Subsequently, it was added to the train subset the ISIC macroscopic images [32] and the training and validation subsets of Set D and Set M. The final configuration of the dermoscopic and macroscopic datasets can be seen in Table 2.

### 3.2. Methodology

#### 3.2.1. Generation of Macroscopic Images

CycleGAN was introduced in 2017 by Zhu et al. [12], this framework is a technique used for the adversarial “unsupervised” training of image translation between two different domains. The most notable capacity is the ability to transform within two domains without using paired data. This lack of pixel-to-pixel correspondence happens in the macroscopic and dermoscopic images, even if images correspond to the same lesion as each acquisition implies different illumination, resolution and distance to the lesion. The framework consists of two mapping functions G:X−>Y and F:Y−>X in conjunction with the respective adversarial discriminators, Dx and Dy, that distinguishes x from F(y) and y from G(x), respectively. These duos are trained with an adversarial loss ($L_{GAN}$) in association with a cycle consistency loss ($L_{cyc}$).

**Figure 2:**
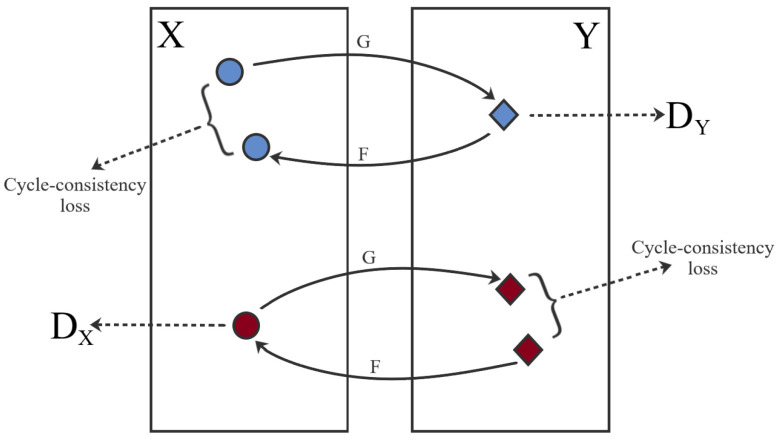
CycleGAN framework and training strategy. Blue—Forward cycle-consistency loss; Red—Backwards cycle-consistency loss.

In this work, the objective function used in the computation of the $L_{GAN}$ was a combination of the sigmoid cross-entropy between the generated images and an array of ones, and the L1 loss interpolated from the generated image and the target image. Nevertheless, the $L_{GAN}$ alone is not enough to produce images of quality, since it imposes the generated output to be in the desired domain, but does not promote the recognizability between the source and target domain. To enable this mode of learning, Zhu et al. [12] proposed $L_{cyc}$. This mechanism, which can be seen in Figure 2, relies on the expectation that if an image is converted to another domain and back again it will be comparable to the original image. This generates two cycle-consistency losses: the forward cycle and the backward cycle.

The full loss is a weighted sum of the adversarial loss of both mapping functions ($L_{GAN}\left(F,D_{X},Y,X\right)$) and $L_{GAN}\left(G,D_{Y},X,Y\right)$) and the cycle consistent loss ($L_{cyc}\left(G,F\right)$), where the $L_{cyc}\left(G,F\right)$ was favoured by a λ=10 as shown in (1).

(1) $$\begin{array}{cc} L(G,F,D_{X},D_{Y}) & =L_{GAN}\left(F,D_{X},Y,X\right)+L_{GAN}\left(G,D_{Y},X,Y\right)+\lambda L_{cyc}\left(G,F\right). \end{array}$$

**Generator Architecture** The architecture adopted is based on the approach described by Johnson et al. [33] in 2016, which was used for neural style transfer and superresolution. This network can be divided into three sections: the encoder, the transformer and the decoder. The encoder is comprised of three sets of padding and convolutional layers followed by instance normalization and the ReLU activation function. The first convolutional layer has a 7 × 7 kernel and stride 1, and the last two have 3 × 3 kernels and stride 2. Afterwards, more representative features are produced with the transformer, which encompasses a series of twelve residual blocks of 3 × 3 kernels and stride 1, instead of the nine residual blocks used in the Johnson et al. [33]. Lastly, the feature maps are expanded by the decoder, which is comprised of two transposed convolutions, with 3 × 3 filters and stride 2, and one output convolutional layer with 7 × 7 kernels and stride 1. This last layer has the typical *tanh* activation function to produce the final image in RGB.

**Discriminator Architecture** For the discriminator, the PatchGAN [34] was used. This CNN looks at a “patch” of the input image and ascertains the probability of being real. This fully connected network is comprised of five convolutional layers with 4 × 4 kernels and stride 2. Each layer is followed by an instance normalization and the Leaky ReLU activation function. The output layer uses a sigmoid function to perform the binary classification between real and fake images.

**Implemention Details** The implementation of the CycleGAN was made with Tensorflow API r1.15 in Python 3.7.3 on an NVIDIA Tesla V100 PCIe 32GB GPU. As training protocol, the one proposed on the original paper [12] was followed. In short, we employ a learning rate of 0.0002 with the weights initialized from a Gaussian distribution ranging from 0 to 0.02. The stopping criteria took into consideration the stabilization of the loss values of the generators, as well as the immutability of the TransMacro and TransDermo images, which were visually analysed during training. In the limiting case in which none of the criteria were satisfied, the CycleGAN model had the final criteria of 1000 epochs. The dataset used in the training and testing of this network is described in Table 2.

#### 3.2.2. Segmentation Network

The segmentation network employed was the optimized encoder-decoder network selected in Reference [10], which used a modified DeepLabV3+ [35]. Initially, the encoder was replaced by a MobileNetV2 [36] reduced with a width multiplier, which allows the manipulation of the input width of a layer, of α=0.35. Additionally, the last convolutional layer was adjusted to a 1 × 1 convolutional layer with a sigmoid activation function, which is more appropriate for a binary segmentation.

Before the training procedure, the images were scaled and resized to 512 × 512 pixels. The following classic data augmentation procedure was applied: the images were randomly flipped vertically and horizontally, randomly transposed, randomly modify brightness, saturation, contrast and hue, and randomly add Gaussian noise. To optimize the network, we followed the procedure from the previous work [10]: the stochastic optimization of the model was performed with the soft Dice Loss (1 − Dice), with a batch size of 4, a 90/10 partition for the training/validation subsets and the Adam optimizer associated with a cyclic learning rate [37].

#### 3.2.3. Evaluation

Although the evaluation the generated images is still an active frontier of research [38], they may be analysed by visual inspection, metrics of similarity and the impact of using them in segmentation can also be calculated.

**CycleGAN evaluation metrics** The visual inspection was based on domain-specific knowledge of intrinsic dermoscopic features, such as pigmented networks and diffused pigmentation, the appearance of macroscopic artefacts, for instance, the surface glare and reflections, and, lastly, the generation of the outward aspect of depth and the modification of the background skin tonality.

To quantitatively evaluate the similarity between the generated and the real images, the Fréchet Inception Distance (FID) [39] was used. This metric measures the distance between the features spaces of specific CNN layer, typically the last pooling layer of InceptionV3 [40], for the real and real and generated images. Since it is based on a distance metric, a lower FID score means that the two analysed distributions are similar. FID is considered a discriminative, robust metric, and evidence shows that it is consistent with human judgement [39,41].

**Segmentation evaluation metrics** For the evaluation of the segmentation results, the six metrics used in the ISIC challenge of 2018 [28] were adopted, namely: threshold Jaccard coefficient (TJA), Jaccard coefficient (JA), dice coefficient (DI), accuracy (AC), sensitivity (SE) and specificity (SP). The TJA is a metric created specifically for the skin lesion segmentation, which introduces the measure of incorrectness. If the value of JA, on each image, is below 65% threshold then JA is considered 0.

## 4. Results and Discussion

### 4.1. Evaluation of the Generated Images

#### 4.1.1. Visual Inspection

A visual assessment was performed to evaluate the outputs’ realism, by identifying the loss or preservation of some dermoscopic structures. The datasets that had both dermoscopic and macroscopic for the same skin lesion were used, namely the EDRA and SMARTSKINS 2014/2015. The same analysis was carried out with images from the ISIC and PH2 test subsets; these images were not used during the training phase of the CycleGAN.

**Figure 3:**
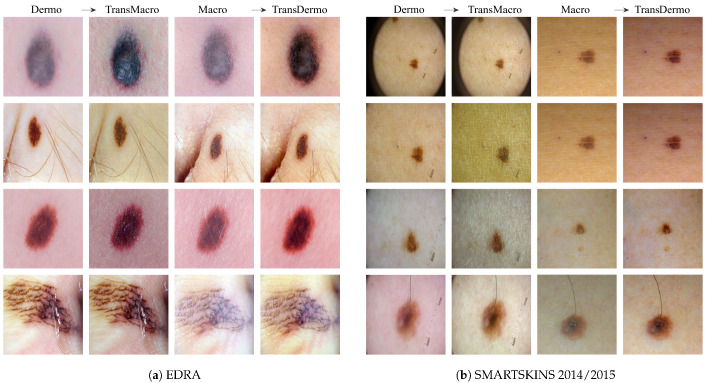
Examples of the translation between domains in EDRA (**a**) and SMARTSKINS 2014/2015 (**b**) tests subsets. For each subfigure, from left to right: pair Dermo→TransMacro and pair Macro→TransDermo.

**EDRA**Figure 3a shows the results obtained from the trained CycleGAN in the test subset of the EDRA dataset—It is possible to compare the macroscopic image to the dermoscopic image translated into the macroscopic domain (TransMacro) and the macroscopic image translated into the dermoscopic domain (TransDermo). In the case of the dermoscopic to macroscopic translation, we can observe that the model was able to generate plausible macroscopic images from dermoscopic images. In particular, several successful transformations unique to the macroscopic domain were obtained, such as: (i) The generation of depth in the TransMacro images, that is, lesion images with nodular aspect from flat dermoscopic images, shown in Figure 3a (row 1); (ii) The expected appearance of surface glare and change of skin tonality due to the absence of polarized light in macroscopic images, which can be seen in Figure 3a (rows 1, 2, and 3); and (iii) The loss of dermoscopic characteristics such as specific pigmentations or diffuse borders, as illustrated in Figure 3a (rows 1 and 2). It should be noted that failure cases occur when there is the presence of gel or ruler markings and round black borders. These three characteristics never appear in macroscopic images, so, understandably, the model could not learn how to address these artefacts. Where there is gel in the lesion, the model only performed slight modifications to the input, as shown in Figure 3a row 4.

However, the inverse transformation seems to lead to unsatisfactory results. One possible explanation is the incapacity of the model of extrapolating the greater details of the dermoscopic images. Considering all the specific details normally obtained in the dermoscopic image, the model was not able to capture sufficient features to solve this task.

**SMARTSKINS 2014/2015** This dataset proved quite challenging for the model due to the dark corners in all dermoscopic images. Figure 3b (row 1) shows this failure case, which usually results in minimal to no changes in the TransMacro result. Since we suspected that the cause of failure was the fact that macroscopic images never have these dark borders, and consequently the generator does not learn how to deal with the characteristic, the dark corners were removed, by cropping the image. This pre-processing step led to a considerable improvement in the results. Figure 3b (row 2), shows the translation of the same dermoscopic image, this time cropped. Here, the model was able to generate an appearance of reflection and modify the skin tonality, which can be seen in Figure 3b (rows 2, 3, and 4). In this dataset, the generation of depth skin lesion can also be seen in Figure 3b (row 4), however not as evidently as in the previous dataset.

In this dataset, an interesting result was obtained in the macroscopic to dermoscopic translation. Figure 3b (row 4) shows the generation of a region with red colouration in the left inferior area of the lesion of the TransDermo image. This colouration, which was not present in the original macroscopic image, is also present in the original dermoscopic image. Another transformation detected, in this image was the accentuation in the contrast of the two brown tonalities of the lesion.

**Figure 4:**
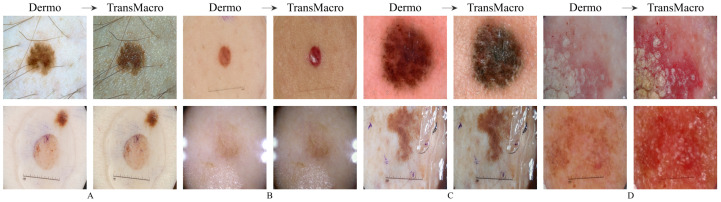
Examples of successful (**top row**) and failure cases (**bottom row**) of translation from the dermoscopic domain to the macroscopic domain in ISIC test subset. The letters A, B, C and D represent pairs of Dermo→TransMacro.

**ISIC**Figure 4 shows the results obtained from the trained CycleGAN in the ISIC test subset. As expected, the best transformations occured in this subset, as the ISIC images represented a large portion of the training dataset. For the most part, the conversions in this dataset can be categorised into four main ones which are shown in Figure 4 in the top row: (i) the appearance of surface glare and reflections; (ii) generation of depth; (iii) loss of dermoscopic structures; and (iv) generation of squamous appearance. Regarding the generation of surrounding skin with colourization and surface glared, typical of the macroscopic domain (Figure 4, pair A, top row), this modification is usually accompanied by the loss on any structure typical of the dermoscopic domain. This loss can be from the diffuse borders, regression structure or pigmented networks (Figure 4, pairs A, B and C, top row). Another frequent modification is the generation of squamous like plaque or nodular appearance (Figure 4 pair D of the top row). The generation of an appearance of depth in the image is also very prevalent (Figure 4, pairs A, B and C, top row).

In the translation of dermoscopic to the macroscopic domain, there are several noticeable limitations, as shown in Figure 4 (bottom row). The appearance of artefacts such as black frames, dark corners, gel substances, intensive illuminations spots, ink marks and air bubbles can lead to translations with no noticeable modifications (Figure 4, pairs A, B and C, bottom row). Another main concern is the transformation to an uncharacteristic lesion which does not resemble any specific domain. Normally these translated images have a reddish tone or complete loss of definition (Figure 4, pair D, bottom row).

**Figure 5:**
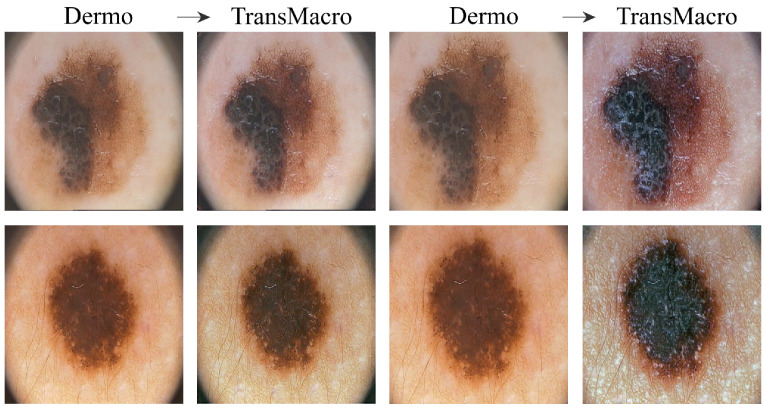
Examples of successful (column 1 and 2) and failure cases (column 3 and 4) of translation from the dermoscopic domain to the macroscopic domain in the PH2 test subset. From Left to right: pair Dermo→TransMacro, cropped pair Dermo→TransMacro.

**PH2**Figure 5 shows the results obtained from the trained CycleGAN in the PH2 test subset. In order to obtain reasonable results, it was necessary to crop the image to remove the dark corners just as it was done in the SMARTSKINS 2014/2015 dataset. After this pre-processing step, the most successful transformations were the change in the tonality of the surrounding skin, the addition of reflections, the darkening of the lesion and some elevation of the lesions. However, it should be noted that the results were not as satisfactory as in the previous test subsets.

#### 4.1.2. Fréchet Inception Distance Results

The FID metric was used to ascertain the feasibility of using the CycleGAN to translate between the macroscopic and dermoscopic domains. For this purpose, the distance between three pairs of domains was analysed: (i) Macro/Dermo, between the macroscopic and dermoscopic original images; (ii) Macro/TransMacro, between the original macroscopic image and the dermoscopic image translated into the macroscopic domain; (iii) Dermo/TransDermo, between the original dermoscopic image and the macroscopic image translated into the dermoscopic domain. Since the first pair, Macro/Dermo, compares original images, it can be considered our reference value. If the FID value of the other pairs is lower than the reference value, it means that the translation led to an approximation of the translated images to the target domain, implying a transfer of characteristics between domains.

This change can also be directly compared using the variation ratio (VR), VR = $\frac{\mathrm{Reference}\mathrm{Value}-\mathrm{Value}}{\mathrm{Reference}\mathrm{Value}}$, which will directly compare the change of similarity between the original domains (reference value) and the translated domains. Table 3 presents the computed FID scores, using the official implementation in TensorFlow [39], on the test subsets of EDRA, SMARSTSKINS 2014/2015, PH2 (set D) and ISIC (set D). Since PH2 (set D) and ISIC (set D) do not have a macroscopic image for each dermoscopic image, they were compared with the macroscopic images of the train subset of Dermofit (set M) and SMARTSKINS (set M).

When analysing the FID results, it is possible to confirm several findings reached in the visual inspection. In the EDRA test subset, the FID score of 160.2 between the domains Macro/TransMacro is lower than the value 167.9 between the Macro/Dermo. This shows that the translations of the dermoscopic images to macroscopic was successful and implies that the images gain specific macroscopic characteristic. In contrast, the value between Dermo/TransDermo is much higher, reaching 186.4. When comparing to the reference value, it leads to a negative VR, which indicates that translating the macroscopic images into dermoscopic made the domains more dissimilar. This further corroborates the conclusion of the visual analysis that the macroscopic to dermoscopic translation was not successful.

In the SMARTSKINS 2014/2015 test subset, the macroscopic images were compared with the uncropped and cropped dermoscopic images, as in the visual analysis. Considering that the decay between the reference FID score with the cropped dermoscopic (Macro/TransMacro) is much higher than decay between the reference value using the uncropped dermoscopic images, it can be stated that this preprocessing step improves the results. In fact, the VR of the Macro/TransMacro with cropped dermoscopic is the highest among test subsets. When analysing the other translation (Dermo/TransDermo), the absolute value of the FID score with the cropped dermoscopic (263.6) is lower than with the uncropped dermoscopic (285.6). However, the VR is also lower, which means that the preprocessing did not improve as much the results.

In the ISIC (Set D) test subset, the dermoscopic images were compared with the train images of Set M. Upon translation, the absolute FID score is the lowest of the test subsets (123.7), validating the assessment made in the visual inspection—the best results were obtained in this dataset.

The PH2 test subset was also compared with the train images of Set M. Here, the FID value obtained was the highest absolute value with the lowest percentage of variation, which is consistent with the small changes observed in the visual inspection.

Lastly, the CycleGAN was used to translate the training/validation images from Set D to the macroscopic domain. This led to the creation of the Set M${}_{artificial}$ to augment the macroscopic images. Table 4 shows the results of the FID between the segmentation training datasets set M and Set D and the new generated Set M${}_{artificial}$. The FID score between Set M/set M${}_{artificial}$ (102.4) is much lower than the reference value between Set M/Set D (181.1). This drop leads to the most significant change in the variation ratio between the reference value and the Macro/TransMacro domains. Considering also the low absolute value of the FID score (102.4), it is possible to conclude that the images of Set M${}_{artificial}$ obtain several key features of the macroscopic domain.

### 4.2. Segmentation Results

The final objective of translating the images to the macroscopic domain was improving the model’s segmentation capacity for macroscopic images. Thus, the reduced Mobile DeepLab model was trained with two different datasets, first with the merging of Set M and Set D and, after, with the combined Set M and Set M${}_{artificial}$. The first dataset (Set M + Set D) serves to check if the performance improvement is due to more samples or the addition of the translated images. The datasets had a total of 3230 samples: being Set D + Set M composed of 2/3 of dermoscopic images, while the Set M + Set M${}_{artificial}$ is composed of 2/3 of macroscopic images artificially generated with the CycleGAN.

Table 5 compares the methods described in the related work [10,16,18], which exploit the SMARTSKINS database, with the two aforementioned models. The addition of the translated macroscopic images, Set M + Set M${}_{artificial}$ (Table 5, row 6), outperforms the other methods by a considerable margin. This augmentation technique leads to an improvement even when compared to the classical augmentation methods (Table 5, row 3), to the transfer learning technique (Table 5, row 4) and to the addition of the original dermoscopic images (Table 5, row 5). With an 85.13% TJA and 86.69% JA, our method, that includes the translated macroscopic images, sets a new state-of-the-art performance in the SMARTSKINS database.

For the Dermofit database, there was only one record of the use of this database for segmentation [10], which is compared with our methods in Table 6. In the Dermofit test subset, the addition of artificial macroscopic images (Table 6, row 4) has similar results to the model trained with Set M + Set D (Table 6, row 3). In fact, both models, while outperforming the classical data augmentation (Table 6, row 1), seem to underperform when compared with the transfer learning method (Table 6, row 2) used in Reference [10]. However, it is only possible to make a distinction with the TJA metric, which features a 1.16% decrease. This underperformance means that only four more images were below the 65% threshold in the model trained with the artificial macroscopic images (Table 6, row 4) than with the transfer learning method (Table 6, row 2).

The fundamental reason behind this discrepancy in the results between the two databases can be the melanocytic bias present in most of the databases. Since the past research focused on the distinction between melanocytic lesions [29], almost all databases are heavily biased towards this type of lesions. Nowadays, there has been an attempt to solve this issue, however, this is mainly in dermoscopic datasets [28,29]. Since SMARTSKINS database has this biased distribution of lesions it leads to a significant improvement. However, Dermofit Image Library has a high non-melanocytic percentage of images. Due to this disparity with all other available databases the improvement is not as substantial.

**Figure 6:**
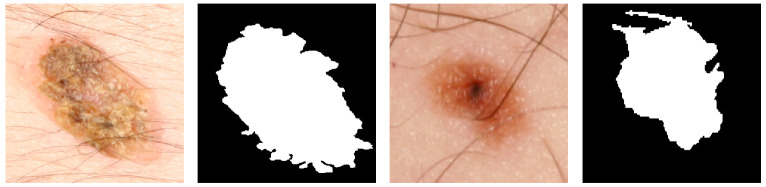
Examples of questionable segmentation labels of the Dermofit Database.

It is of note that in Dermofit questionable ground truth segmentation masks were found as depicted in Figure 6. Both these labels appear to be the result of a segmentation method which over-segmented the lesion due to the hair artefacts present in the images, which is a common occurrence when the removal of the hairs is ineffective [42].

**Figure 7:**
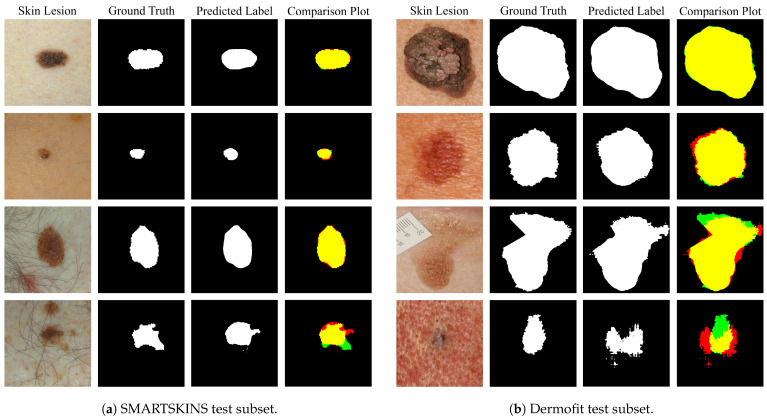
Segmentation results of the Set M + Set M${}_{artificial}$ from the tests subsets. In the comparison images: yellow—true positives; red—false positives; green—false negatives; black—true negatives.

Figure 7a,b show various examples of the predicted segmentation mask of the model trained with the addition of the artificial images, Set M + Set M${}_{artificial}$, compared with the ground truth label. The juxtaposition of the predicted segmentation mask with the ground truth originated a plot which makes it possible to ascertain the viability of the segmentation by colour code. Fundamentally, the yellow represents where both masks overlap (true positives), the red the over-segmented areas (false positives) and the green the under-segmented areas (false negatives). Over the entire test subset, the normalized value of false positive pixels is of 0.12 for the Dermofit and 0.032 for the SMARTSKINS dataset and the normalized value of false negatives is of 0.04 and 0.007, respectively.

When analysing the results in detail when using the model that includes generated macroscopic images from each of the test subsets, it is possible to observe that all segmentation results are almost identical in shape to the ground truth when dealing with melanocytic lesions with high contrast with the surrounding skin and no artefacts present (Figure 7a,b row 1). This high performance is maintained with lesions with low contrast and uneven pigmentation (Figure 7a,b row 2). Furthermore, if we observe Figure 7a,b row 3, it is possible to see that the model is able to correctly segment in the proximity of regions with the red colouration, with hairs and superimposed artefacts. However, the model seems to underperform when the lesion presents a dysplastic form, with the hair artefact, and with heavily porous skin and low contrast (Figure 7a,b row 4).

## 5. Conclusions

Nowadays, with skin cancer’s ever-growing incidence, developing a robust system capable of assisting physicians in cancer screening is of paramount importance. The ubiquitous spread of smartphones combined with deep learning models’ robustness makes mobile teledermatology a possible tipping point for the early diagnosis of skin lesions. However, this has been hindered by the small size of macroscopic datasets available.

In this paper, we tackle the challenge of improving the macroscopic skin lesion segmentation performance by effectively taking advantage of the large dermoscopic datasets available. Using a CycleGAN, it was possible to translate between the two domains and generate natural-looking artificial macroscopic skin lesions. Furthermore, we demonstrate the quality of the generated images quantitatively using the FID score. This analysis confirmed most of the visual inspection conclusions and demonstrated the artificial macroscopic images’ fidelity.

Regarding the use of macroscopic artificial images to improve the segmentation capacity, it was demonstrated the overall effectiveness of this method. In both available databases, there was an improvement when comparing with classical data augmentation method. As future work, we should consider to examine the segmentation masks of the Dermofit Image Library, in order to correct them, however it should be noted that this segmentation should be done preferable by experts.

Undeniably, the assessment of the images by an expert dermatologist would be interesting to further evaluate the relevance of the generated medical data. However, this is not critical since the final objective is to improve the segmentation model’s performance.

Further work is needed to overcome the melanocytic bias of the databases. This could begin with the collection of new non-melanocytic macroscopic images. However, when taking into consideration the arduousness of collecting healthcare data, the applications of differentiated augmentation techniques should be studied. This can include the analysis of loss functions that represent more faithfully our design goals, such as condition-specific losses or texture-focused losses as in Reference [22]. Other improvements can also be made to the generator architecture of the CycleGAN framework since its goal is the translation of style, and it has limited translation capacity in form transfiguration.

## Figures and Tables

**Table 1:** Overview of available segmentation macroscopic and dermoscopic databases and separation into train/validation and test subsets.

Set	Database	No. Images (Type)	No. Train/Val.	No. Test
set M	Dermofit	1300 (MD)	1036	264
	SMARTSKINS	80 (MP)	39	41
set D	ISIC	2594 (De)	1994	600
	PH2	200 (De)	160	40

set M—set of macroscopic images; set D—set of dermoscopic images; No-Number of images, De-Dermoscopic images, MD-Macroscopic images acquired with a digital camera, MP-Macroscopic images acquired with a mobile phone.

**Table 2:** Formation of dermoscopic and macroscopic datasets and separation into train and test subsets.

Database	Train		Test	
	MD∖MP	De	MD∖MP	De
EDRA	802	802	209	209
SMARTSKINS 2014/2015	295	148	56	28
PH2 (Set D)	-	160	-	40
ISIC (Set D)	-	1994	-	600
Dermofit (Set M)	1036	-	-	-
SMARTSKINS (Set M)	39	-	-	-
ISIC Archive	104	-	-	-
Total	2158	3057	242	869

De-Dermoscopic images, MD-Macroscopic images acquired with a digital camera, MP-Macroscopic images acquired with a mobile phone.

**Table 3:** Fréchet Inception Distance (FID) and Variation Ratio (VR) results in the test subsets of EDRA, SMARTSKINS 2014/2015, PH2 and ISIC.

Domains	EDRA		SMARTSKINS 2014/2015				ISIC (Set D)		PH2 (Set D)	
			Uncropped		Cropped				Cropped	
	FID	VR	FID	VR	FID	VR	FID	VR	FID	VR
Macro/Dermo (reference value)	167.9		331.7		294.2		180.9		292.9	
Macro/TransMacro	160.2	0.05	285.1	0.15	177.5	0.40	123.7	0.31	285.7	0.02
Dermo/TransDermo	186.4	−0.11	285.6	0.14	263.6	0.10	-		-	

**Table 4:** Fréchet Inception Distance (FID) and Variation Ratio (VR) results between set M, set D and set M${}_{artificial}$.

Domains	Segmentation Sets	FID	VR
Macro/Dermo (reference value)	Set M/Set D	181.1	
Macro/TransMacro	Set M/set M${}_{artificial}$	102.4	0.43

**Table 5:** Comparison between the proposed methods and related studies using the SMARTSKINS database. The best result, for each of the metrics in the present table, is identified in boldface.

Method	TJA	JA	DI	AC	SE	SP
Adaptive Thresholding [16]	-	81.58	-	97.38	-	-
Gossip Network [18]	-	-	83.36	-	-	-
Reduce Mobile DeepLab (Macroscopic) [10]	78.51	82.64	90.14	98.96	95.40	99.15
Reduce Mobile DeepLab (Transfer Learning) [10]	78.04	82.21	89.89	98.90	96.05	99.09
Our Method (Set M + Set D)	78.27	82.58	90.22	98.88	98.39	98.89
Our Method (Set M + Set M${}_{artificial}$)	**85.13**	**86.69**	**92.74**	**99.18**	**96.36**	**99.32**

**Table 6:** Comparison between the proposed methods and related studies using the Dermofit Image Library database. The best result, for each of the metrics in the present table, is identified in boldface.

Method	TJA	JA	DI	AC	SE	SP
Reduce Mobile DeepLab (Macroscopic) [10]	72.97	80.26	88.26	93.51	87.56	96.86
Reduce Mobile DeepLab (Transfer Learning) [10]	**75.46**	**81.03**	**88.79**	**93.78**	**89.68**	**96.13**
Our Method (Set M + Set D)	74.42	80.88	88.75	93.53	90.74	95.14
Our Method (Set M + Set M${}_{artificial}$)	74.30	80.90	88.62	93.54	88.91	95.84

## Data Availability

International Skin Imaging Collaboration (ISIC) challenges datasets: Publicly available dataset was analyzed in this study. This data can be found here: https://challenge.isic-archive.com/data. PH2 dataset: Publicly available dataset was analyzed in this study. This data can be found here: https://www.fc.up.pt/addi/ph2%20database.html. SMARTSKINS segmentation and 2014/2015: The data presented in this study are available on request from the corresponding author. The data are not publicly available due to privacy reasons. Dermofit image Library: Restrictions apply to the availability of these data. Data was obtained from The University of Edinburgh and are available at https://licensing.edinburgh-innovations.ed.ac.uk/i/software/dermofit-image-library.html with the permission of The University of Edinburgh. EDRA: Restrictions apply to the availability of these data. Data was obtained from DS Medica srl and are available at http://www.dermoscopy.org/atlas/order_cd.asp with the permission of DS Medica srl based in Milan in Viale Monza 133 - CF. and VAT number 12676030153.
